# Supplementary material for: Distant sequence regions of JBP1 contribute to J-DNA binding
Source: Life Sci Alliance. 2023 Jun 16;6(9):e202302150. doi: 10.26508/lsa.202302150 (PMC10276184; doi:10.26508/lsa.202302150)
Supplement: Supplementary file 1 [file LSA-2023-02150_TableS1.docx]

**Table S1 - Bitclust occupation of the protein models used as input for HADDOCK docking studies of DBD-JBP1 with J-DNA and the occupation of the HADDOCK clusters of the resulted models.**

Structure models are shown in Supplemental Figure S5A-F.

|  | **Bitclust occupation (%)** | **HADDOCK occupation cluster 1 (%)** | **HADDOCK occupation cluster 2 (%)** | **HADDOCK total number of generated clusters** |
| --- | --- | --- | --- | --- |
| **Cluster 1** | 67 | 51 | 24 | 5 |
| **Cluster 2** | 22 | 61 | 15 | 6 |
| **Cluster 3** | 4 | 76 | 8 | 5 |
| **Cluster 4** | 39 | 28 | 23 | 9 |
| **Cluster 5** | 10 | 46 | 19 | 9 |
| **Cluster 6** | 15 | 69 | 14 | 5 |
