# Supplementary material for: Distant sequence regions of JBP1 contribute to J-DNA binding
Source: Life Sci Alliance. 2023 Jun 16;6(9):e202302150. doi: 10.26508/lsa.202302150 (PMC10276184; doi:10.26508/lsa.202302150)
Supplement: Supplementary file 2 [file LSA-2023-02150_TableS2.docx]

**Table S2 - Thermal stability of DBD-JBP1 proteins used in fluorescence polarization assays.**

| **Protein** | **Tm (0.25 mg/ml protein)** | **Tm (0.5 mg/ml protein)** |
| --- | --- | --- |
| JBP1-DBD-Wt | 35.3 °C | 34.5 °C |
| JBP1-DBD-E437A | 27.5 °C | 26.3 °C |
| JBP1-DBD-H440A | 37.2 °C | 36.3 °C |
| JBP1-DBD-R448A | 38.7 °C | 37.7 °C |
| JBP1-DBD-N455A | 34.4 °C | 33.3 °C |
