# Supplementary material for: Distant sequence regions of JBP1 contribute to J-DNA binding
Source: Life Sci Alliance. 2023 Jun 16;6(9):e202302150. doi: 10.26508/lsa.202302150 (PMC10276184; doi:10.26508/lsa.202302150)
Supplement: Supplementary file 3 [file LSA-2023-02150_TableS3.docx]

**Table S3 - Affinity of DBD-JBP1 for J-DNA and T-DNA.**

N.d. stands for ‘not determined’.

| **Protein** | **K_d_ J-DNA (nM)** | **K_d_ T-DNA (µM)** | **Specificity (K_d_ T-DNA/K_d_ J-DNA)** |
| --- | --- | --- | --- |
| JBP1-DBD-Wt | 17 ± 1.8 | 178 ±37 | 10749 |
| JBP1-DBD-E437A | 30 ± 2.8 | n.d. | n.d. |
| JBP1-DBD-H440A | 57 ± 8.8 | 432 ± 91 | 7599 |
| JBP1-DBD-R448A | 8190 ± 137 | 1274 ± 1103 | 156 |
| JBP1-DBD-N455A | 6845 ± 844 | 413 ± 126 | 60 |
