# Supplementary material for: Distant sequence regions of JBP1 contribute to J-DNA binding
Source: Life Sci Alliance. 2023 Jun 16;6(9):e202302150. doi: 10.26508/lsa.202302150 (PMC10276184; doi:10.26508/lsa.202302150)
Supplement: Supplementary file 4 [file LSA-2023-02150_TableS4.docx]

**Table S4 - HADDOCK cluster occupation of the docking results of the DBD-JBP1 new crystal structure with J-DNA as shown in Figure 5.**

AU stands for arbitrary unit.

|  | **HADDOCK occupation count, (%)** | **HADDOCK score (AU)** |
| --- | --- | --- |
| **Cluster 1** | 151, (87%) | -86.6 |
| **Cluster 4** | 5, (3%) | -72.3 |
| **Cluster 3** | 8, (4% | -65.4 |
| **Cluster 2** | 26, (14%) | -61.0 |
