# Supplementary material for: Distant sequence regions of JBP1 contribute to J-DNA binding
Source: Life Sci Alliance. 2023 Jun 16;6(9):e202302150. doi: 10.26508/lsa.202302150 (PMC10276184; doi:10.26508/lsa.202302150)
Supplement: Supplementary file 5 [file LSA-2023-02150_TableS5.docx]

**Table S5 - Affinity of JBP1, truncated JBP1 and DBD-JBP1 for J-DNA.**

| **Protein** | **K_d_ J-DNA (nM)** |
| --- | --- |
| JBP1 | 6.4 ± 0.7 |
| Δ23-JBP1 | 20.8 ± 0.9 |
| Δ38-JBP1 | 31.2 ± 2.4 |
| DBD-JBP1 | 20.7 ± 2.2 |
